# On Typhus

**Published:** 1806-09

**Authors:** 


### *To the Editors of the Medical and Physical Journal.*

Gentlemen,

After an absence of more than six years, the Typhus Fever has again made its appearance in this neighbourhood. The disease was no sooner recognized, than I betook myself with earnestness to the volumes of Dr. Currie. I rely implicitly on every fact therein advanced, I follow him, in so far as respects the treatment of this disease, I follow him in every step, and have hitherto had no reason to repent me of my guide; than whom, perhaps, the history of medicine does not furnish a character, more enlightened, more respectable, or more lamented in his death. I have

I have at present seen fifteen cases of this disease, all of which are now recovered, or recovering; and cold water, in one way or other, was applied to the surface of all. In one case indeed, exclusive of these, the complaint was immediately arrested in its progress by the affusion of cold water. Two brothers were already down; a third, had alternate chills and heat, head-ache, and disturbed sleep. It was late in the evening when I saw him; he had a burning skin, and pulse 110. I had him taken out of bed, placed in a tub, and poured two buckets full of cold water over him. When I called the next morning he had left his bed, and was walked out of the house; in the evening again, at his own suggestion, the same remedy was renewed; when I next saw him he appeared to be perfectly recovered, and from that time ailed not.

The affusion was employed in three other cases, with the evident effect of cutting short the disease; it was begun with about the third day, and by the seventh, the sufferers were convalescent. It was practised also on a fifth patient, where it did not so directly succeed; but that he went through the disease quicker, and, comparatively, with much less suffering, I have no doubt. The remedy was not used until about the sixth day of the fever, by which time, we may presume, the morbid actions and associations were too firmly established to yield so immediately as in the other cases. When I first saw him there was every appearance of a dangerous disease; he had a violent head-ach, great debility, was low in bed, with muttering, and startings of the tendons; tongue and fauces so dry as to prevent him almost from articulating; pulse 120, heat 104. The affusion was used night and morning only, being as often as I could see him; I repeatedly sat down by him for ten minutes after the water had been thrown over him, and always found his pulse about 90, heat 94; and he was still and composed for some time after. We went on in this way for five days, when he began to perspire violently, and lost his superabundant heat during that process; when an indication now arose, we had recourse to ablution, and he was about fifteen days from the date of his illness, before he could be said to be much better; from which time he recovered.

A boy, about ten years of age, was taken with the complaint; his heat never rose so high as in the former case, we therefore did not proceed with him beyond ablution; and the disease came on so insidiously, that even *that*

was was not applied until it had made some progress; he had now great stupor upon him, but was frequently roused as from a dream, delirious, and was with difficulty composed again; he was costive with distended belly, twice he appeared to be a good deal relieved by the operation of a suitable dose of pulv. scam. cum calomel; his head was shaved with the intention of putting on a blister, but its burning feel induced us for the present, to wash it only with vinegar and water; in a day or two it was covered with petechiæ, and a few were scattered on his body; his nose, which he was constantly fretting, bled a little, for two or three days together; from this time he became sensible, began to look up, and has had a slow, but perfect recovery.

Deafness in the progress of the complaint came on in all; the debility attending was very great; the erect posture in several of the patients could not be supported even for a few minutes on a nurse's knee. The progress of emaciation was very rapid, so that they, who when last observed were looking well, rose out of bed again mere skeletons, and on their first tottering steps out of doors, made a fearful impression on some of their more opulent neighbours.

In the two cases which I have given more in detail, cold seemed very evidently to be the remote cause of the fever; exposure to contagion directly, or indirectly, could not be made out; from whence, I am at present with those, who think that this agent, under certain circumstances and conditions of the body, may produce fever of debility.

All the patients took bark and wine, and opiates and opening medicines, as occasion required; but I am inclined almost to attribute every thing to the reduction of the temperature of the body by cold water; and I most gladly and confidently join in the opinion, that the true remedy for typhus is simple, and at hand; that it dwells in every fountain, travels in every stream, and murmurs in every rill.

A PRACTITIONER *in Derbyshire.*

*August* 8, 1806.

